# Utilization, financial outcomes and stakeholder perspectives of a re-organized adult sickle cell program

**DOI:** 10.1371/journal.pone.0236360

**Published:** 2020-07-24

**Authors:** Robert Rousseau, Daniel F. Weisberg, Jack Gorero, Vivek Parwani, Janis Bozzo, Kathleen Kenyon, Constance Smith, Joanna Cole, Susanna Curtis, Ariadna Forray, John D. Roberts

**Affiliations:** 1 Northeastern University Bouvé College of Health Sciences, Boston, MA, United States of America; 2 Icahn School of Medicine at Mount Sinai, New York, NY, United States of America; 3 Hartford Hospital, Hartford, CT, United States of America; 4 Yale School of Medicine, New Haven, CT, United States of America; 5 Yale New Haven Health, New Haven, CT, United States of America; 6 Yale New Haven Hospital, New Haven, CT, United States of America; Technion - Israel Institute of Technology, ISRAEL

## Abstract

In 2011 Yale New Haven Hospital, in response to high utilization of acute care services and widespread patient and health care personnel dissatisfaction, set out to improve its care of adults living with sickle cell disease. Re-organization components included recruitment of additional personnel; re-locating inpatients to a single nursing unit; reducing the number of involved providers; personalized care plans for pain management; setting limits upon access to parenteral opioids; and an emphasis upon clinic visits focused upon home management of pain as well as specialty and primary care. Outcomes included dramatic reductions in inpatient days (79%), emergency department visits (63%), and hospitalizations (53%); an increase in outpatient visits (31%); and a decrease in costs (49%). Providers and nurses viewed the re-organization and outcomes positively. Most patients reported improvements in pain control and life style; many patients thought the re-organization process was unfair. Their primary complaint was a lack of shared decision-making. We attribute the contrast in these perspectives to the inherent difficulties of managing recurrent acute and chronic pain with opioids, especially within the context of the imbalance in wellness, power, and privilege between persons living with sickle cell disease, predominantly persons of color and poor socio-economic status, and health care organizations and their personnel.

## Introduction

Yale New Haven Hospital (YNHH) and Yale University have a longstanding tradition of service and research related to sickle cell disease (SCD) [1–16]. Yet, in 2010 medical leadership recognized that YNHH care for adults living with SCD needed improvement. This arose from three sources: (1) YNHH average hospital length of stay (ALOS) for adults living with SCD was the highest among the six highest SCD volume hospitals in the state [17]. (2) Frequent complaints by patients and concerns expressed by providers and nurses indicated widespread dissatisfaction. (3) YNHH leadership had received complaints from community minority and patient advocacy organizations. The purpose of this report is to describe our strategy to improve SCD care for adults, its implementation; outcomes in terms of utilization, costs, and stakeholder responses; and to discuss some implications of the experience.

## Methods

### Description of the program

YNHH is a large tertiary academic medical center located in an urban setting in the New York—Boston corridor with a strong affiliation with the Yale School of Medicine. The nearest acute care hospitals with emergency departments (ED) are 9, 11, 19, 24 and 29 miles away; the nearest with an organized adult sickle cell program (prior to 2017) is 42 miles away.

In 2011 YNHH organized a small group of administrators and physicians to make recommendations regarding the re-organization of care for adults living with SCD. Recommendations included:

1. New physician leadership, increased advanced practice provider (APP) support, increased social work support, and the introduction of psychiatry support.
2. Re-locating inpatient services from multiple medical floors to a new Adult Sickle Cell Unit with new nursing leadership and new nursing staff recruited for and trained in the care of adults living with SCD.
3. Re-assignment of inpatient attending responsibilities from multiple general medicine attendings, often working with housestaff, to hematologists, working exclusively with APPs.

These recommendations were implemented in 2011 and early 2012. In July 2012, a newly recruited medical director began working with others to further re-organize the program.

#### Approach to pain management with opioids

The long ALOS was viewed as an indicator of excessive utilization of acute care services and excessive reliance upon parenteral opioids for the management of pain. A programmatic approach to pain was developed according to the following principles:

1. Most adults living with SCD experience chronic pain punctuated by acute pain exacerbations [18].
2. For many or most patients, optimal pain management involves the use of opioids, as well as other drugs and modalities [19].
3. Chronic pain should be managed at home with non-parenteral opioids, as well as other drugs and modalities [19]. Acute pain usually should be managed at home as well, but occasionally management requires parenteral opioids administered in an acute care setting, such as an infusion center, ED, or hospital. A major programmatic goal was to convert as much utilization as possible from unscheduled acute care visits with occasional providers to scheduled outpatient visits with providers engaged in the long term care of the patients.
4. Whenever a large group of patients with any disease are treated with opioids, a fraction will develop dysfunctional opioid related behaviors [20–23]. These include seeking opioids for reasons other than pain, such as to cope with anxiety, depression, or insomnia; this has been referred to as “chemical coping”. Many or most of these patients are not aware of the dysfunctional nature of their behaviors or the underlying motivations.
5. The responsibility of providers, and institutions, is to provide pain relief while maintaining vigilance for dysfunctional opioid related behaviors, and, when appropriate, to place limits upon a patient’s access to opioids, sometimes despite complaints of poorly controlled pain.
6. This professional and institutional responsibility inevitably will be addressed imperfectly as: (i) Pain is subjective, and cannot be assessed objectively [19]. (ii) Assessment of opioid misuse also is subjective, as evidenced by the lack of expert consensus upon its identification [21, 22, 24, 25].
7. Management of pain in SCD is complicated by race relations in America: whereas SCD primarily affects persons who self-identify as African-American or Latino, groups that are socio-economically disadvantaged and subject to discrimination, health care professionals largely come from non-disadvantaged ethnic groups and backgrounds, and hospitals are powerful institutions. Decisions by those in power and patients’ assessments of those decisions may be affected by racial bias and perceptions of racism [26–31].
8. Thus, pain management in SCD often is fraught with conflict. The best professional and institutional approach to minimize conflict is to strive for clinical consistency within a comprehensive approach to pain.

#### Implementation of our approach to pain management with opioids

We undertook to improve inpatient pain management while incorporating a number of approaches intended to reduce length of stay. In this we partnered with YNHH hospitalists, who were the admitting providers in most cases, to provide appropriate and consistent approaches to pain management. The previous standard of care had been intravenous bolus opioid administration, which was associated with frequent bedside conflict between patients and providers and nurses about doses, dosing delays, and, sometimes, the rate of intravenous bolus administration. In retrospect, doses often were inadequate. We introduced patient controlled analgesia (PCA), initially as an alternative, and, subsequently, largely to the exclusion of intravenous bolus opioid therapy. Almost all established patients had a personalized care plan available to admitting providers in an easily accessible part of the electronic medical record. This provided reliable, up-to-date information about a patient and specific recommendations regarding pain management. Recommended initial PCA demand doses ranged from morphine 1 mg to hydromorphone 8 mg, a ~ 50-fold range in potency according to estimates of opioid equivalence [32]. We generally continued any outpatient long acting pain medications, but, in order to avoid inadvertent overdosing, we did not provide opiods by continuous infusion. In order to reduce bedside conflict, we did not use a nurse-managed bolus feature of the PCA pump.

We instituted a new approach to inpatient oral administration of opioids, which we call an “oral tier”, in which a dose of a short acting opioid is scheduled for administration every 3 hours, and additional linked PRN orders allow for administration of additional amounts if the patient reports moderate or severe pain, respectively. (Subsequently, we discovered a literature precedent for this [33–35] and published a description of our approach [36].) The scheduled dose, which the patient might refuse, assured that the patient experienced regular pain assessments and opportunities to request oral pain medication; thus, it avoided the delays, frustration, and potential conflict associated with PRN dosing. A typical order set might involve a scheduled dose of morphine 30 mg every 3 hours and PRN doses of morphine 30 mg PRN moderate pain and morphine 60 mg PRN severe pain, yielding options for the patient to take morphine 0, 30, 60, or 90 mg every 3 hours.

Rather than switch from PCA to oral medications, we continued PCA as we initiated an oral tier, typically on the first or second morning after hospitalization. The combination of PCA and oral tier provided patients with considerable flexibility in the timing and amount of opioid received. This allowed us to create an expectation that the pain treatment regimen would be discussed once daily on morning rounds, eliminating many requests to return to the bedside later in the day to re-discuss pain management. Further, this allowed us to create an expectation that revisions of pain management plans by after hours clinicians should be uncommon. We routinely conducted morning rounds as a multi-disciplinary team, potentially including a physician, an APP, an LCSW, a nurse manager and/or nurse, a pharmacist, and a chaplain. Rounding as a group reduced recalls to the bedside to clarify patient, or staff, misunderstanding or miscommunication.

We considered a goal of immediate, complete pain control to be unrealistic, and we considered most of our patients to be experienced with pain treatment. Thus, on rounds our queries focused less upon reports of pain severity and more upon whether the patient thought that they had access to enough pain medication. Although our hospital required regular nursing pain score assessments and documentation, we rarely referred to pain scores on rounds. As pain improved, we instructed patients to maximize use of the oral tier and minimize use of PCA. Low PCA use signaled readiness for discharge to both patients and providers.

We coupled these approaches to pain management with clear expectations that most hospitalizations for pain would be measured in days, not weeks, and a willingness to set limits. Some patients would continue to use PCA to the exclusion of the oral tier, rendering assessment of readiness for discharge impossible. Some patients reported continuing pain and poor pain control in a manner that suggested to us that hospitalization for parenteral opioids could continue indefinitely, that we actually were dealing with chronic pain or chemical coping or some other misuse problem related to opioids or hospitalization. Whenever possible, we offered patients a range of medically acceptable options, and, once offered, we honored their choice. If we thought a specific approach was indicated, we simply stated the plan. For example, we might inform the patient of a planned discharge date a few days hence, and taper and discontinue PCA while encouraging the patient to utilize the oral tier. In these situations, we might explain to the patient that we had two concerns, pain and parenteral opioid misuse, and that at that moment we were more concerned about the latter; alternatively, we might stop inquiring about pain on rounds. Our experience was that while continuing an oral tier we could taper and discontinue PCA in almost all patients in 3 days or less without precipitating withdrawal symptoms. Occasionally a patient would refuse to be discharged, in which case we might declare that all pain medications would be stopped at a certain day and time, and the patient was free to leave at any time. At all times we attempted to focus upon choices among options that we considered acceptable, and not to engage in arguments.

At some point during a hospitalization, we might interview a patient about their approach to pain management at home prior to presenting to the ED. Our goal was to encourage flexible use of home pain meds in order to attempt to control pain at home.

We discharged patients with a scheduled return to clinic in less than two weeks, and we informed patients of our expectation that they would be engaged in outpatient care.

The previous standard of care for the inpatient management of opioid-induced pruritus had been intravenous bolus diphenhydramine, which also was associated with conflict at the bedside about delays in dosing and patient requests for more rapid intravenous administration. Although we had viewed diphenhydramine as a treatment for opioid-induced pruritis, we learned that some of our patients sought rapid bolus administration for anxiolytic or euphoric effects. Previous attempts to convert patients to oral anti-pruritics had been sporadic and ineffective. We picked a date several weeks hence at which all inpatients able to swallow and absorb oral antipruritics would be switched from intravenous to oral administration; we provided health care personnel with a script for bedside conversations that focused upon the available options for anti-pruritic treatment; and we created an expectation among patients and health care personnel that this change would occur and would be permanent. Requests for intravenous bolus diphenhydramine became rare.

Personalized care plans contained recommendations regarding ED management. Often these care plans were created in the patient’s presence, providing an opportunity for patient input. The vast majority of plans specified a decision regarding hospitalization following three parenteral opioid doses in the ED. When our patients made us aware of long wait times in the ED, typically greater than 60 minutes, we partnered with ED leadership in an effort to reduce these. Although initially successful, improvements unfortunately were not sustained. Our patients also made us aware of lapses in pain control in the handoff between ED and hospitalist providers and transfer from the ED to a hospital bed. In order to address this, we partnered with ED providers, nurses, and pharmacists to initiate PCA in the ED upon a decision to hospitalize. This was very successful.

As our patients and providers became familiar with our approach, we began to use the personalized care plans to limit inappropriate utilization. For a patient with repeated ED visits and hospitalizations but no engagement in outpatient management, for example, a care plan might read: “This patient continues to use the ED and hospital for pain, but has failed to engage in our outpatient program for pain management. We should stop enabling this inappropriate utilization of health care resources. Always evaluate for evidence of problems other than pain. For pain, in the absence of objective evidence of acute illness (for example, fever or a new chest x-ray infiltrate), do not treat pain with opioids by any route; do not admit. Discharge from the ED with instructions to call the hematology clinic for an appointment. In the presence of objective evidence of acute illness, we recommend the following treatment for pain”.

### Establishing a comprehensive care approach

YNHH leadership shared a vision that the program should provide a comprehensive approach to the care of the all adults living with SCD who sought care at the institution. Patients were referred to the hematology clinic for ongoing care, largely to the exclusion of primary care clinics or clinics staffed by trainees. In the hematology clinic a primary care or medical home model was adopted in which an effort was made to care for both the special needs of adults living with SCD and the usual health maintenance needs of a predominantly young adult population. Initially, the clinic practice was shared by a small number of hematologists who shared the vision of the newly recruited medical director and a dedicated APP who was continuously available during clinic hours for urgent visits. Subsequently, the medical director assumed responsibility for most patients in clinic as well as rounding on inpatients most days. A psychiatrist was embedded in the hematology clinic so that patients could see both a medical and psychiatric provider during a single clinic session. A dedicated outpatient LCSW was continuously available during clinic hours to provide patients with emotional and logistical support. We had access, albeit limited, to outpatient infusion facilities so that patients could receive hydration, pain treatment, and transfusions on an outpatient basis. When patients were referred to other subspecialists, special efforts were taken to coordinate care. A primary focus of clinic visits was to enable the home management of chronic and most episodes of acute pain. Almost all patients with chronic pain and most patients with intermittent acute pain had access to outpatient opioids (oral and/or transdermal), as well as other pain medications. The range of outpatient opioid use was very large, ranging up to ~ 1200 mg oral morphine equivalents per day. We adopted a policy of prescribing naloxone for emergent reversal of opioid overdose for every patient with a prescription for home opioids. We recommended acetaminophen and prescribed non-steroidal anti-inflammatory drugs frequently. We prescribed adjunctive medications for pain such as anticonvulsants or drugs described as muscle relaxants rarely. Although we did not prescribe selective serotonin reuptake inhibitors or selective norepinephrine-serotonin reuptake inhibitors for pain management, many of our patients received these agents through our program’s embedded psychiatrist.

### Establishing expectations regarding home opioid medications

We established a number of expectations regarding home opioid medications. Patients receiving any opioid prescriptions were seen at least every 6 months; patients receiving prescriptions for both short and long acting opioids were seen at least every 2 months. When appropriate, patients were seen much more frequently, including weekly. Failure to attend clinic could result in suspension of opioid prescribing until a visit occurred. Opioid refills were not available after hours. Patients were instructed that it was their responsibility not to run out of pain medication; if they were using their pain medications faster than anticipated, they were to call and, if required, appear for an urgently scheduled clinic visit. Patients were instructed that we expected to be their sole source of opioid prescriptions. The Connecticut Prescription Monitoring Program was accessed regularly to assess adherence to this expectation. Patients were instructed that their medications should not be shared or sold and should be stored in a secure place. Patients were advised that replacement prescriptions for medications lost or stolen would be uncommon. Our intent was that patients receiving opioid prescriptions undergo urine drug testing at the time of the initial prescription, at least yearly thereafter, and more frequently if we had concerns about inappropriate utilization of opioids or illicit drugs. Urine was tested for the presence of opioids with concurrent documentation of the most recent time of ingestion, the presence of opioids not prescribed by us and presumably prescribed by others or obtained through illicit sources, and absence of drugs of abuse such as cocaine and phencyclidine; we generally ignored evidence of illicit marijuana use. (We published our patients’ self-reported marijuana use elsewhere [37]. And, in a non-hospital role, we successfully partnered with community based organizations focused upon SCD to petition the state to add SCD to the list of qualifying conditions for medical marijuana. Subsequently, we made patients aware of this alternative to illicit marijuana and certified patients for medical marijuana upon request, with rare exceptions.) We developed a number of approaches to facilitate meeting our expectations. Some patients for whom we had determined that further dose escalation was not appropriate would continue to run out of opioid medications early. In such cases, we might issue multiple prescriptions for an appropriate amount of medication to be dispensed on an every-other-day or even a daily basis; or, we might engage visiting nurse services to deliver a few days supply of medications on a frequent basis. Failure, or repeated failure, to meet our expectations would lead to a suspension of opioid prescribing for a defined period of time measured in months. Patients with substance use disorders and chronic pain might be referred to a local substance abuse treatment program where they might qualify for observed daily single dose methadone administration.

We held weekly multi-disciplinary team meetings with equal time devoted to programmatic issues and individual patients.

### Program evaluation

We tracked utilization and costs using administrative clinical and financial data from our hospital cost accounting software systems, initially Allscripts®, and later, StrataJazz®. Data were downloaded from StrataJazz® into a Microsoft Access® database for analysis and uploaded into Microsoft Excel® for data display. In order to assess whether changes observed at YNHH might reflect changes ongoing regionally and potentially unrelated to our program re-organization, we performed an interrupted time series analysis comparing YNHH inpatient days with those of an affiliated community hospital located ~ 15 miles away.

In the second year of the re-organization we reviewed our experience with 122 patients for whom we believed we had sufficient data to characterize their opioid use. This review involved patient-by-patient assessments conducted by medical and psychiatric providers, nurses, and LCSWs in face-to-face meetings (without the patient present) supplemented with chart reviews when indicated.

Inpatient unit registered nurses and emergency department physicians and inpatient were surveyed using custom-designed, web-based surveys that used a Likert scale response format.

We assessed patient perceptions and satisfaction with a custom-designed telephone survey conducted by a hospital volunteer with no other affiliation with the program; patient responses were anonymized as promised by the volunteer. The volunteer elicited both categorical responses in a Likert scale format and additional comments that were captured in interview notes.

Serendipitously, a medical student had conducted a research project involving in-depth interviews with high utilizing patients just prior to initiation of the program re-organization [38]. We invited him to re-interview the same patients about one year later.

We partnered with the Community Health Network of Connecticut (CHNCT), the administrative services organization for Medicaid in our state, to evaluate medical resource utilization of our patient Medicaid members using CHNCT claims data. Data were securely transferred from CHNCT to us in Microsoft Excel® files, then loaded into a Microsoft Access® database for analysis. We used Microsoft Excel® and Tableau® for data display.

#### Ethical review

Interviews with high utilizing patients were conducted as approved by the Yale institutional review board (IRB). All other activities were undertaken as a part of our quality improvement efforts and not submitted for IRB review. The IRB reviewed a draft of this manuscript and concurred with this approach.

### Statistics

Results are described using descriptive statistics, including p values calculated with Stata®. No formal hypotheses for testing for statistical significance were declared.

## Results

### Patients and health care personnel

**Fig 1:**
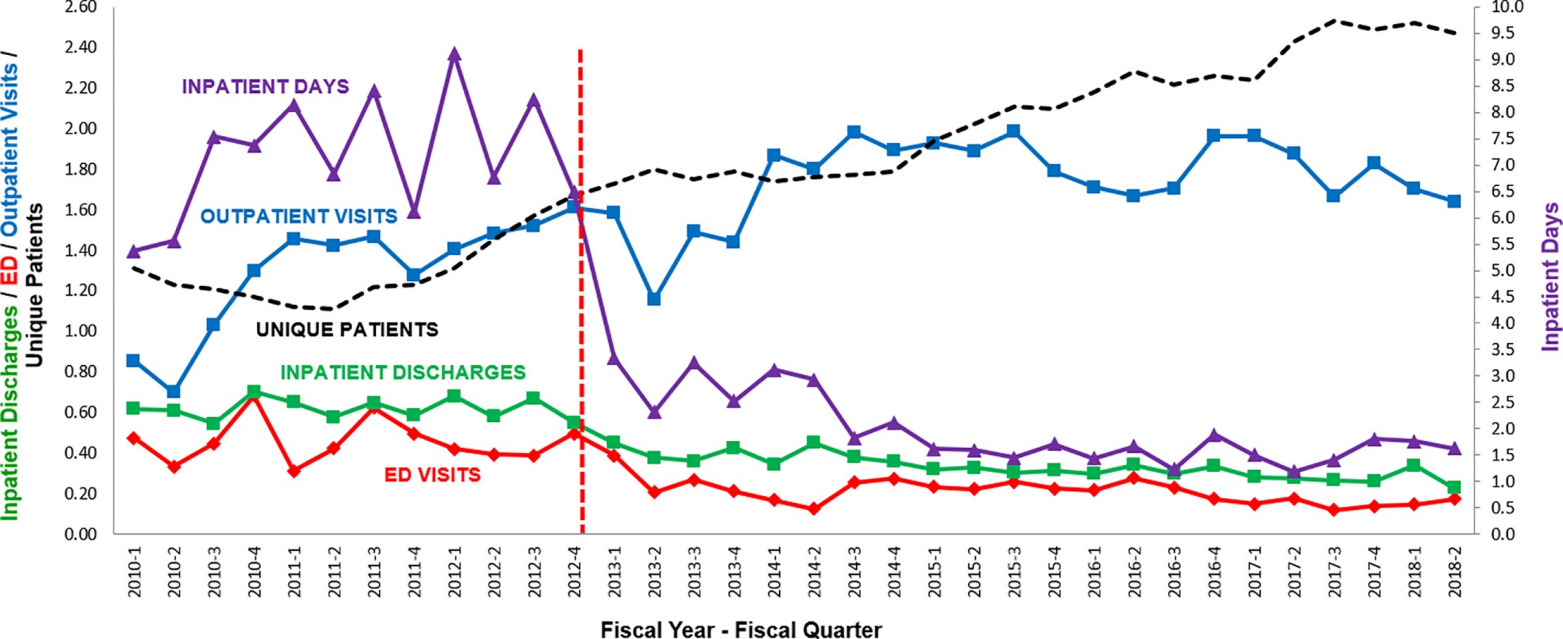
Patients and utilization. YNHH health services utilization–inpatient days, outpatient visits, inpatient discharges, and ED visits—by adults living with SCD from 2010–2018. Outpatient visits includes hospital-based clinics only, including infusion center visits. Inpatient discharges is a surrogate for hospital admissions. Utilization is calculated as utilization events per unique patient per year. Unique patients are re-defined and enumerated on a quarterly basis as any patient 21 years or older with a principal or secondary diagnosis of sickle cell disease (ICD-9 282.60–282.69, 282.41, 282.42; ICD-10 D57.00—D57.219, D57.40—D57.819; not including sickle cell trait, ICD-9 282.5; ICD-10 D57.3) with any encounter (ED, hospital, clinic) within the previous 4 quarters. Unique patient numbers have been divided by 100 to accommodate the vertical scale. The vertical red line indicates the arrival of the new medical director.

We re-define our patient population on a quarterly basis as any patient 21 years or older with a principal or secondary diagnosis of SCD (ICD-9 282.60–282.69, 282.41, 282.42; ICD-10 D57.00—D57.219, D57.40—D57.819; not including sickle cell trait, ICD-9 282.5; ICD-10 D57.3) at any encounter (ED, hospital, or hospital based clinic or infusion center), within the previous year. The population has increased from ~ 150 patients in fiscal year (FY; July 1 through June 30) 2012 to ~ 240 in FY2017 (Fig 1). Patients were about 60% women/40% men with a median age of ~ 31 years. In accordance with YNHH racial/ethnic criteria, patients self identified as Black/African American 86%, Other 9%, White 4%, and Asian 1%, and as Non-Hispanic 91% and Hispanic 9%. Most patients lived in greater New Haven, but some patients lived as far as ~ 50 miles away. About 55% had only Medicaid insurance, ~ 25% Medicare and Medicaid, and ~ 20% commercial or other insurance or were self-pay. In our state patients with Medicaid do not have co-pays or deductibles associated with ED, hospital, or office-based care.

Our SCD team presents as racially and ethnically diverse with approximately half persons of color including Black or African American and Hispanic. Our medical director presents as White, non-Hispanic.

### Utilization

A dramatic change in YNHH health care utilization by adults living with SCD was apparent within months of arrival of the medical director and was sustained for several years (Fig 1; S1 Table). Utilization was tracked as events per patient per year. Comparing FY2012 and FY2017, there was a 63% decline in ED visits from 1.5 to 0.56; a 53% decline in hospitalizations from 2.2 to 1.1; and a 79% decline in inpatient days from 27.3 to 5.7. Average length of stay fell 55% from 12.3 to 5.5 days, with dramatic declines in stays ≥ 10 days (S1 Fig). Outpatient visits increased 31% from 5.4 to 7.1 visits. Infusion center visits, which are included in the outpatient visit totals, could not be enumerated precisely due to limitations of our accounting systems. Nevertheless, these visits declined following the re-organization as we eliminated a pre-existing practice of scheduling infusion center visits for pain more than one day in advance and availability decreased as an increasing cancer patient population largely filled the infusion center with scheduled visits.

Comparison of YNHH inpatient days with those of an affiliated community hospital located ~ 19 miles away did not suggest that changes reflected some ongoing regional change (S1 Document).

### YNHH costs

Changes in YNHH utilization were associated with dramatic reductions in ED and hospital direct costs for caring for adults living with SCD. Comparing FY2012 and FY2017, these costs fell from $4.3 million to $1.6 million (− 63%) despite a greater than 60% increase in the number of unique patients served. Annual clinic costs increased from $0.2 million to $0.7 million (+ 215%). Annual net direct cost fell from $4.5 million to $2.3 million (− 49%). We did not attempt to calculate indirect costs, overhead, or revenue; and, due to changes in accounting systems, we could not calculate changes in contribution margin for the described time period.

### Safety

Our approach to pain control was safe. We had no inpatient or outpatient deaths attributed to opioids. Use of naloxone to reverse opioid-induced respiratory depression in inpatients occurred less than once a year. In five years one patient was transferred to an intensive care setting for possible opioid-induced respiratory compromise. One patient’s child accessed the patient’s medications and overdosed, resulting in life-threatening complications.

### Qualitative assessment of patient opioid use

Among 122 patients, we identified 19 patients (16%) whom we were concerned might be engaged in opioid diversion, that is, sharing or selling. Three patients were women whom we suspected were coerced to divert by domestic partners. In one case our suspicions were confirmed forensically; otherwise, our suspicions were circumstantial. Among the 122 patients, 62 (51%) underwent urine drug testing at least once from February 2013 through September 2014. Of the 19 patients about whom we had concerns regarding opioid diversion, 9 (47%) tested positive on at least one occasion for use of illicit drugs other than marijuana (cocaine, phencyclidine, MDMA [ecstasy]), and 1 additional patient (5%) tested positive for prescription opioids not prescribed by us. Over time the numbers of patients about whom we had concerns about diversion decreased.

We identified 29 patients (24%) for whom we thought a major aspect of their opioid use was related to chemical coping on an inpatient basis with parenteral opioids (15 [52%]), or an outpatient basis with oral/transdermal opioids (2 [7%]), or both (12 [41%]). A minority of these patients were engaged in other dysfunctional behaviors: we suspected 9 (31%) of diversion including 4 (14%) with evidence of illicit drug use and 1 (3%) who tested positive for opioids not prescribed by us.

### Adult sickle cell unit nurse assessment

**Fig 2:**
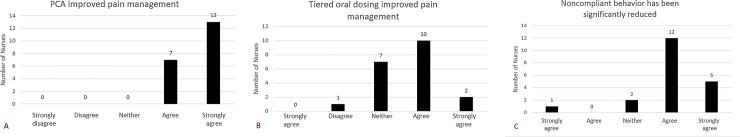
Adult sickle cell unit nurse assessment. Selected adult sickle cell (inpatient) unit registered nurse survey results. Response options are presented in the figures. Numbers are numbers of respondents choosing each response. Questions: (A) “Patient Controlled Anesthesia (PCA) improved pain management.” (B) “Tiered oral dosing improved pain management.” (C) “Noncompliant behavior by patients has been significantly reduced with the implementation of integrated care plans”.

In March 2014 we surveyed registered nurses working on the adult sickle cell unit. Of 26 nurses invited to participate anonymously, 20 completed the survey (S2 Document). Nurses perceived that pain was better controlled with the new approaches of PCA and an oral tier (Fig 2A and 2B). They also perceived that the re-organization was successful in reducing conflict (Fig 2C).

### ED physician assessment

**Fig 3:**
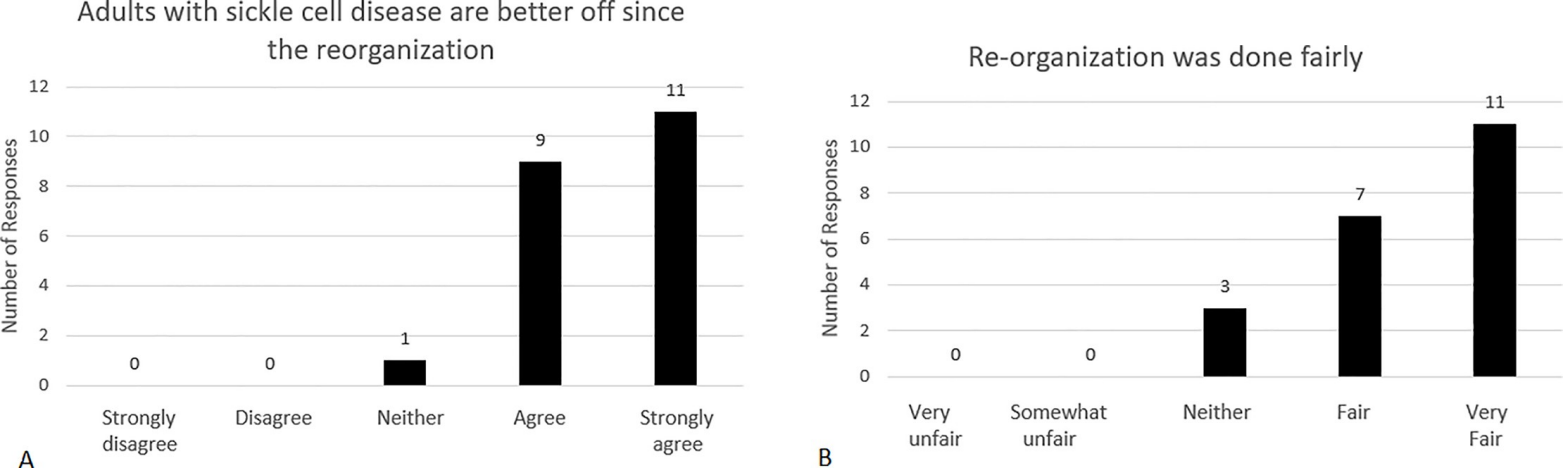
ED physician assessment. Selected emergency department physician survey results. Response options are presented in the figures. Numbers are numbers of physicians choosing each response. Questions asked: (A) “Do you think adults with sickle cell disease are better off since the re-organization?” (B) “Do you think the re-organization was done fairly?”

ED physicians rapidly adopted the practice of looking for and following recommendations of personalized care plans. In March 2016 we anonymously surveyed a sample of ED physicians with experience before and after the program re-organization (S3 Document). Of 24 physicians invited to participate, 21 completed the survey. Large majorities reported that patients were receiving better care and that the re-organization had been fair to patients (Fig 3A and 3B).

### YNHH administration assessment

YNHH administration highlighted the successes of the program re-organization at several internal, regional, and national conferences, at an accreditation visit by the Joint Commission of the American Hospital Association in February 2017, and in the 2017 Annual Report [39].

### Patient response: Utilization of other facilities

**Fig 4:**
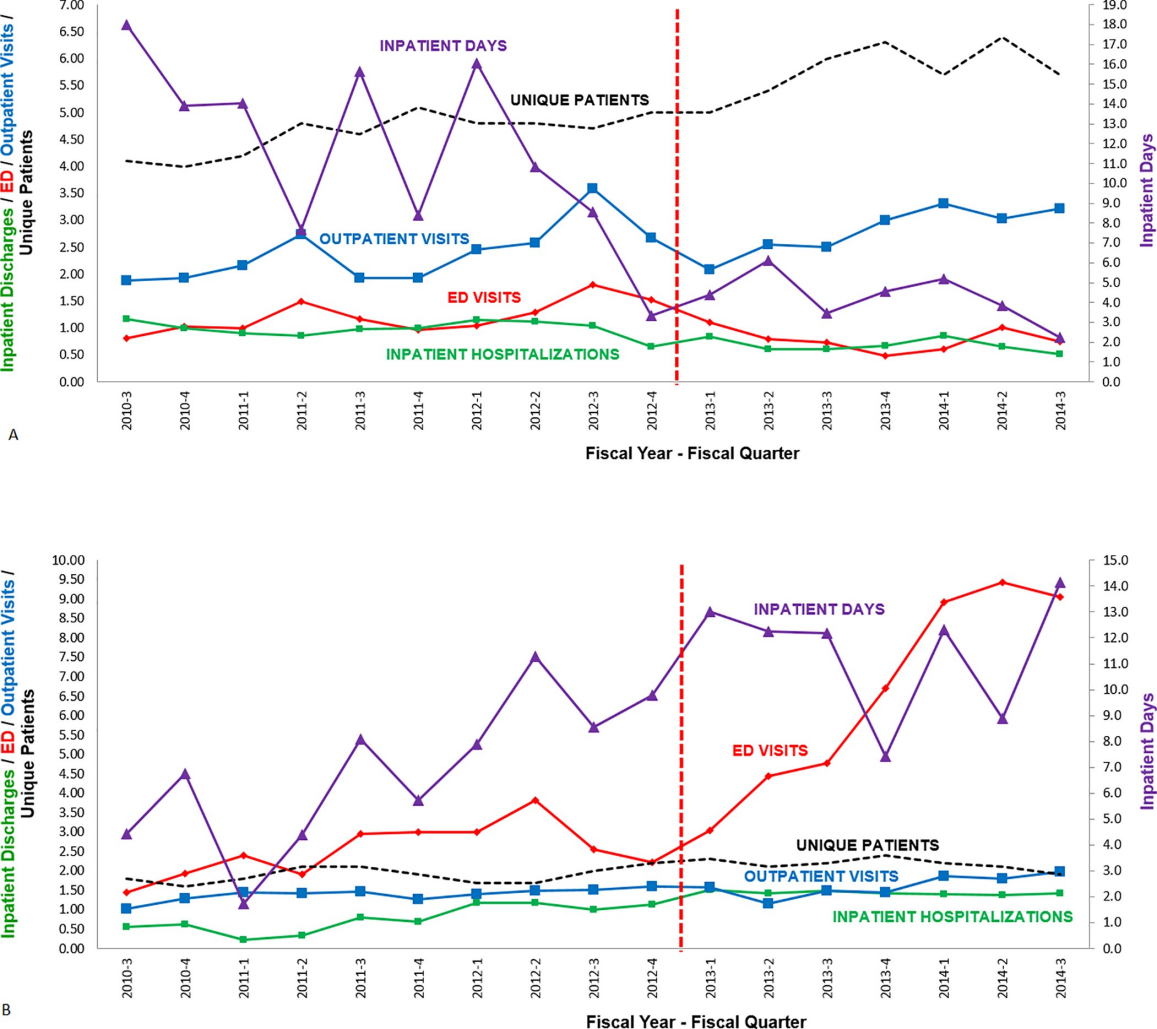
YNHH and non-YNHH utilization by patients with any YNHH utilization. Health services utilization by adults living with SCD with only Medicaid insurance and any YNHH utilization from FY2010-3 through FY2014-3. Unique patients were counted on a quarterly basis as patients with any utilization within the previous 4 quarters; these counts have been divided by 10 to accommodate the vertical scale. Utilization is calculated as utilization events per unique patient per year. Inpatient discharges is a surrogate for hospital admissions. The vertical red line indicates the arrival of the medical director. Note differences in vertical axes. (A) YNHH utilization. (B) Non-YNHH utilization. For all utilization categories, pairwise difference of differences comparison of FY2010-3 through FY 2012–4 versus FY2013-1 through FY2014-3, p < 0.002. For data see S2 Table.

We were aware anecdotally that some patients were seeking care elsewhere subsequent to the program re-organization. We partnered with CHNCT to analyze both YNHH and non-YNHH utilization by patients with only Medicaid insurance (OMI). In 2015 we identified OMI patients who had any YNHH utilization from the 3^rd^ quarter of FY2010 through the 3^rd^ quarter of FY2014 (Fig 4A, 4B; S1 and S2 Tables), and we tracked their utilization during this time. At any given time CHNCT data is limited to patients with active Medicaid insurance. The more remote the time period, the greater the likelihood of Medicaid discontinuation in the interval. The apparent number of patients with ongoing YNHH utilization gradually increased from ~ 40 to ~ 60. This increase probably represented both an actual increase in the ongoing number of utilizing patients, similar to the increase in utilizing patients without regard to insurance status (Fig 1; S1 Table), and an artifactual increase due to Medicaid discontinuations. The apparent number of patients with ongoing non-YNHH utilization gradually increased from ~ 17 to ~ 22. This increase probably also represented both an actual increase in ongoing non-YNHH utilization and an artifactual increase due to Medicaid discontinuations. With these caveats in mind, changes in YNHH utilization of OMI patients were similar to those of the entire YNHH population (compare Figs 1 and 4A; S1 and S2 Tables). Changes in non-YNHH utilization, however, were largely inverse to changes at YNHH with increases in ED visits, hospitalizations, and hospital days. (compare Fig 4A and 4B; S2 Table). Thus, it appears that a minority of patients chose to seek acute care services elsewhere rather than, or in addition to, seeking YNHH care during and following the re-organization.

### Conflict

A motivation for the re-organization was to address conflict between patients and medical and nursing personnel over patient care, which largely was focused upon pain management and utilization of acute care services (ED and hospital). This conflict was exacerbated by inconsistent attitudes and practices among hematology attendings. Upon his arrival, the new medical director began to establish a consistent approach that frequently involved setting limits as described in Methods: Description of the Program. This initially resulted in an increase in conflict that was apparent on a daily basis on rounds and at other times throughout the day and night. We identified a number of themes in patient complaints: Our interventions would not be or were not effective (“I know my body”; “I know what works”). We did not understand or empathize with their condition (“You don’t know my pain”). We were following our rules rather than individualizing care (“We are not all the same”). We were not engaging in shared decision-making (“You are not listening to me”). We were racist (“You wouldn’t do this if I/we weren’t black”). Some patients used profane language. Some patients would fail to engage during rounds, for example, by burying their head in a pillow, not answering questions, and refusing to be examined. Some patients would call their mother in the midst of the interview to complain or solicit support, and the mother might request to speak with the provider to advocate for her adult child. Medical and nursing personnel found these interactions professionally challenging and personally unpleasant. Sometimes the rounding team would conclude that further discussion would not be productive and walk out of a patient room as a patient continued to state their case. Occasionally the rounding team would engage hospital security to address disruptive behavior. On one occasion the medical director was physically assaulted by a patient.

Over the course of several months, however, conflict decreased dramatically. For example, whereas at the outset patient relations personnel were called to the unit by patients on a nearly daily basis, eventually such calls occurred less than monthly.

### Patient assessment: Survey responses

In 2015 we sought to telephone survey 49 patients whom we thought would have had significant outpatient and inpatient experiences before, during, and after the program reorganization. From July through December, 13 patients could not be contacted; 4 declined with 3 commenting that they no longer considered themselves to be YNHH patients; and 32 patients, 9 men and 23 women, were surveyed. Interviewees were engaged and apparently frank as, although the survey questions could be answered in less than 10 minutes, interviews frequently lasted 15 to 30 minutes.

**Fig 5:**
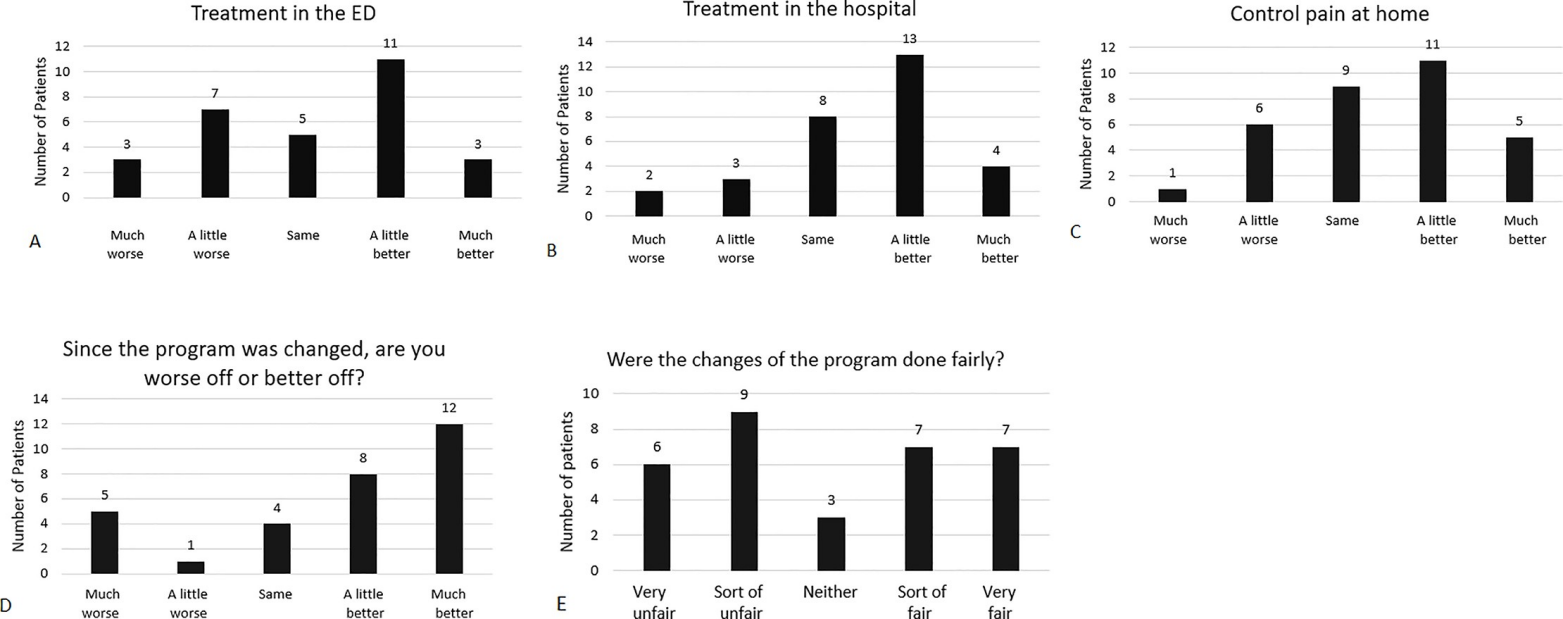
Patient survey responses. Selected results of patient satisfaction survey. Response options are presented in the figures. Numbers are numbers of patients selecting each response. Up to three patients did not provide responses to some questions. Questions: (A) “Since the program was changed, has your treatment in the emergency department changed? Is your treatment in the emergency room:”. (B) “Since the program was changed, has your treatment in the hospital changed? Is your treatment in the hospital:”. (C) “Since the program was changed, has your ability to control your pain at home changed? Is your ability to control your pain at home:”. (D) “Since the program was changed, are you worse off or better off?”. (E) “Were the changes of the program done fairly? Were they:”.

About one-half of patients reported treatment improvements, whereas about one-fifth reported the opposite. Specifically, 48%, 57%, and 50% of patients reported improvements in their treatments in the ED, hospital, and ability to control pain at home, respectively, whereas 31%, 15%, and 22% reported diminishments (Fig 5A, 5B and 5C). Overall, 62% of patients reported that they were better off following the re-organization of the program, whereas 19% reported that they were worse off (Fig 5D). Of interest, 47% reported that the change process was unfair, whereas 44% reported that it was fair (Fig 5E). The response differences between global status and fairness are not statistically significant by conventional criteria (Fisher’s Exact Test, p = 0.08), but we believe that they are clinically meaningful when considered in context. The survey data is consistent with qualitative comments from both the survey and in-depth interview data (see below). Further, the survey response to the fairness question may be an underestimate of patient perceptions of unfairness due to a framing effect [40], as the question was immediately preceded by the question about over all status that elicited largely positive responses. The difference between emergency physicians’ and patients’ assessments of fairness was significant by conventional statistical criteria (Fisher’s Exact Test, p < 0.003).

Notes of patient comments were taken during the interview and subsequently analyzed for common themes. Eight patients made comments about the inpatient adult sickle cell unit; 6 were positive and 2 negative. Eight patients commented that they had inadequate access to our outpatient infusion facilities for the treatment of pain crises. These comments stimulated hospital leadership to identify space for a dedicated outpatient infusion facility located on the adult sickle cell inpatient unit. Seven patients made positive comments about our inpatient nursing staff, with no negative comments. Ten patients commented upon our doctors in general with 7 positive and 3 negative comments; 6 commented upon the medical director by name with 1 positive, 4 negative, and 1 ambivalent comment; one of our other doctors was commented upon positively by 2 patients. Nine patients commented upon individualization of patient care: 5 praised our individualized approach, whereas 4 complained that we applied new policies without regard for individual needs. Five patients complained that the program re-organization was conducted without patient input.

### Patient assessment: in depth interviews

Previously, we had recruited 8 adults living with SCD from the inpatient service and conducted in-depth interviews with these patients as well as 8 family members or significant others and 5 providers identified by these patients as most important in their care [38]. In 2014 these patients were re-contacted and 7 of the original 8 agreed to participate in follow-up interviews. Qualitative analysis of interview transcripts was conducted as described previously. The following are summary findings, which are accompanied by patient quotes in quotation marks and italics; quotes are from 5 patients.

Patients recognized that the process of change was difficult:

• “*Once I turned sixteen*, *they just started administering the medication*, *like however many hours*, *straight through the IV…*. *when you’re used to one thing and that’s what’s been working for you*, *it’s kind of hard to get on board with something different”*.

New policies intended to reduce inpatient days were considered unfair:

• “*I hadn’t gotten out the bed yet to walk around the floor, but yet, I’m going home the next day…I learned to live with it… but I hate it.”*

• “*I have to deal with the thought of [them] making me leave, you know, before I’m better, and …that’s just more stress and make the pain and everything worse.”*

Patients described enduring more pain at home:

• “*I just have to get through it, you know, go home, and you know, take a bath in Epsom salt, relax… [now] I don’t go until I’m like in a full blown crisis”.*

Similarly, new policies intended to promote attendance at scheduled outpatient visits were considered unfair or burdensome, although perhaps with some benefit:

• “*As long as I been going here, I never heard them say ‘if you don’t make it to this appointment, we’re not gonna give you no medicine.’ That’s wrong.”*

• “*Who wants to come to the doctor every two weeks*? *But*, *if I’m out the hospital*, *I’m good*”.

Patients commented positively on opportunities to discuss changes in their outpatient pain medications and to engage with the psychiatrist, social worker and nurse practitioner.

Patients recognized other improvements. They described themselves as:

• *“less sick”*.

One patient described an increased sense of responsibility for his disease management:

• “*I used to come here just to get my medicine, and I leave. And I miss the follow-up appointment…. Now, I’m here every week, and every Monday, every Wednesday….I respect myself more than what I used to do”.*

Another patient described a change in her mood:

• “*I’m not down as much, you know, it’s like, when I [was] in the hospital…I’m feeling depressed sometimes”.*

Patients recognized that they had new opportunities to spend time with their families, and some described pursuing work or further education.

• “*The whole 21 to 35, my grandmother took care of my son. You know, she raised him. And [I] was absent…. When the new doctor came in, [my son is] in the twelfth grade. So now, I get to go to prom, you know, see all those good things.”*

Patients had mixed comments about the medical director:

• “*I know he don’t pull out the bull crap… The old doctors…you could play with their minds—this is what I need*, *… I’ll see you next round*, *might be a month …*. *He’s not going for it… he want us to see life”*.

• “*He think we just up here abusing the drug. That’s how he sees it.”*

• “*I been coming here since I was a baby….but then [the program director] came in and made all these changes and things were totally different…. things have changed and are starting to get better…I’ve started to trust him, he’s started to trust me.”*

## Discussion

Pain is the clinical hallmark of SCD, and opioids are the mainstay of pain treatment [19]. Whenever people are systematically exposed to opioids, it is inevitable that some fraction of them will seek and use opioids inappropriately. Previous estimates of the prevalence of opioid misuse among adults living with SCD generally have ranged up to ~ 10%, which is not significantly different from estimates for adults at large [41]. Previous estimates largely antedate the description of chemical coping as a form of opioid misuse. Estimates of the prevalence of chemical coping among patients using opioids for other diagnoses are ~ 20% [22, 23], which is similar to our estimate of 24% in our patients. Thus, we believe that concern about opioid misuse is very appropriate when caring for persons living with SCD. At the same time, we recognize that all of these estimates are much lower than estimates of the prevalence of opioid misuse among persons living with SCD by health professionals, which range up to ~ 50% [26, 29]. Indeed, these unfavorable attitudes have led to studies of interventions specifically designed to improve health professionals’ attitudes towards persons living with SCD [42, 43]. Thus, as stated in our principles, we believe that the management of pain, opioids, and opioid misuse in persons living with sickle cell disease is subjective, challenging, and imperfect.

Prior to the re-organization, we believe, as has been suggested by others [44], that we were enabling patients to accommodate to living in the hospital on parenteral opioids for extended periods of time largely for chronic pain or chemical coping. It is unlikely that such a situation would have evolved if patients had to pay co-pays or deductibles to access this care, but most did not. We dramatically shifted health care resource utilization from the ED and hospital to the outpatient clinic through investment in a program that focused upon management of most pain at home through patient engagement in a clinic practice staffed by providers with a long term commitment to their care. A motivation for our program re-organization was ongoing conflict between patients and health care personnel over pain management, and implementation of new program policies involved a transient increase in conflict. Some patients responded to this by seeking episodic care from ED and hospitalist providers at surrounding facilities. Nevertheless, we believe that the outcomes were positive: Most patients reported improvements in pain management both in acute care settings and at home; patients were spending much less time in the hospital; some patients volunteered that this had allowed them to pursue fuller lives; overt conflict was dramatically reduced. If one accepts that much of the prior utilization represented management of chronic pain or chemical coping with parenteral opioids, then the dramatic reduction in ED and hospital utilization also represents a dramatic reduction in inappropriate use of parenteral opioids. We remain concerned, however, that our outpatient prescribing practices continue to enable chemical coping by some patients.

Very high utilization of acute care services by a small fraction of adults living with SCD is a common theme of the SCD outcomes literature [45–49]. Providers from several institutions have reported successful initiatives to reduce high utilization. Many of these focus upon creation of a day hospital or infusion center for the administration of parenteral opioids, as well as other treatments, outside of the ED or an inpatient unit [44, 50–54]. This approach generally has reduced ED visits, hospital days, and associated costs, and presumably decreased patient inconvenience and increased patient satisfaction. On the other hand, we see day hospital utilization for pain not only as costly and inconvenient as compared with home management, but also as a potential marker of inappropriate reliance upon parenteral opioids for chronic pain and/or chemical coping. None of the cited reports address the impact of a day hospital upon trends in parenteral opioid use.

Others have described reductions in ED visits and hospital days and associated costs by focusing upon high utilizing patients [53, 54]. Such initiatives would seem to have limited impact upon the quality of care for the majority of an institution’s patients living with SCD.

Others have described programmatic re-organizations similar to ours and with similar impacts upon acute services utilization [44, 50, 51, 53, 54]. A common feature of all of these reports is that they do not mention conflict. We find this surprising as our experience involved a lot of conflict; and, anecdotally, in our conversations with other clinicians, including some who have reported programmatic re-organizations, several, but not all, acknowledged conflict that was not discussed in their reports. We speculate that others are reluctant to report conflict because it doesn’t fit their vision of what good care should look like.

Although not a part of this report, but consistent with reports by others [50, 51], we believe that re-focusing patient care upon the clinic has allowed us to be more effective in implementing best practices in the care of adults with SCD such as utilization of hydroxyurea; reduction of unnecessary red blood cell transfusions; utilization of chelating agents for patients with iron overload; pursuit of an appropriate, extended vaccination schedule; promotion of effective contraception when women do not desire pregnancy; promotion of screening for and treatment of sickle cell retinopathy and nephropathy; screening for and treatment of chronic hepatitis C; and promotion of regular dental care. Further, we screen patients for psychiatric and psychological disorders and refer patients to an embedded psychiatrist for medication and counseling. And, we screen patients for biopsychosocial impediments to health and well-being and address those problems as best as we can with dedicated LCSWs.

All of these efforts should be viewed within the context that access to continuing care is a serious, unaddressed problem facing adults living with SCD [55]. It is likely that many or most adults living with SCD in the United States do not have ready access to providers or facilities with a commitment to comprehensive continuing care. No wonder, then, that health care utilization by adults is skewed toward acute care services.

It is gratifying that almost two-thirds of our surveyed patients reported that they were better off following the program re-organization. It obviously is of concern that a large minority of these same patients reported that the approach taken was unfair. It is unclear to what extent this grievance was responsible for one-fifth of surveyed patients reporting that they were worse off following the program re-organization. Although without intending to dismiss this concern, it perhaps is relevant to note that others have suggested that racial differences alone between patients and providers may result in less favorable patient assessments [33]. We propose two opposing interpretations of our findings. Perhaps we were unfair: Perhaps similar improvements in outcomes could have been accomplished with a shared decision-making approach. But we think this is unlikely. We think that fear of uncontrolled pain; chemical coping; an institutional history of enabling accommodation to a dysfunctional lifestyle organized in part around repeated dosing with parenteral opioids; and, in some cases, conventional drug addiction, would have doomed to failure a shared decision-making approach. We accept that accomplishing our objectives required a provider-directed approach, and that a transition period of increased patient-provider conflict was a regrettable but intrinsic part of this approach.

Our program re-organization involved many components, and one might wonder which elements were critical to success. We would propose the following: In adults living with SCD, most pain should be managed at home with medications (and other measures) prescribed within the context of regularly scheduled outpatient visits. This requires an outpatient facility, or practice, that is committed to the longitudinal management of pain, and, ideally, other aspects of SCD, including the medical, psychological, psychiatric, and social aspects. Unfortunately, it is our impression that many communities with significant numbers of adults living with SCD do not have such a facility. Within such a facility, it is important that providers share a similar approach to the use of opioids. This is most easily accomplished when the number of outpatient providers is small. As it is inevitable that ED and hospitalist providers will be larger in number, more diverse in approach, and without any long term commitment to these patients, it is best if the approach to care in the ED and hospital is guided by the outpatient providers. Personalized care plans available to ED and admitting hospitalists is an efficient method for providing this guidance. When patients remain in the hospital for more than a few days, it is best if an outpatient clinician is consulted and largely permitted to direct inpatient management; the alternative is inconsistent care, which fosters conflict and a tendency to allow patients to lapse into dysfunctional behaviors. Access to an outpatient infusion facility for the administration of parenteral opioids, as well as hydration and transfusions, reduces patient inconvenience and may save costs, but the impact of such a facility upon opioid misuse, especially chemical coping, could vary widely depending upon its management. Given the centrality of pain and pain management with opioids to the care of adults living with SCD and the intrinsically subjective nature of that management, creation of an integrated outpatient and inpatient program requires leadership with the authority or persuasiveness to implement change.

## Supporting information

S1 Table (DOCX)

S2 Table (DOCX)

S1 Fig (PDF)

S1 Document (DOCX)

S2 Document (DOCX)

S3 Document (DOCX)
