# Supplementary figures and images for: Utilization, financial outcomes and stakeholder perspectives of a re-organized adult sickle cell program
Source: PLoS One. 2020 Jul 24;15(7):e0236360. doi: 10.1371/journal.pone.0236360 (PMC7380627; doi:10.1371/journal.pone.0236360)

S1 Table

Utilization.


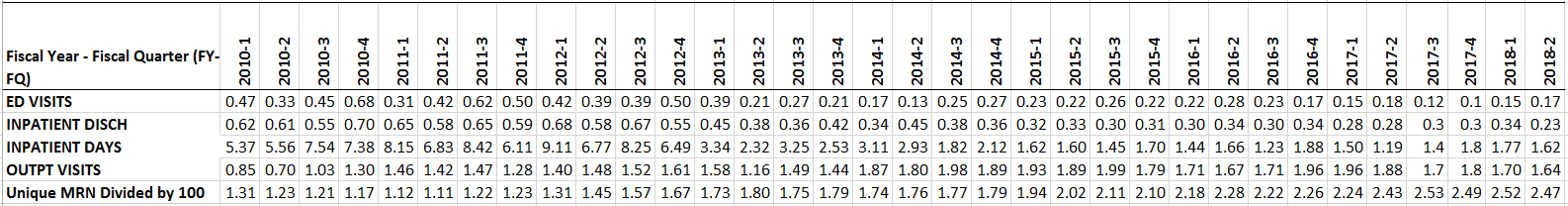


See Fig 1 Legend.

Supplement: S1 Table — (DOCX) [file pone.0236360.s001.docx]

## YNHH - Volume by LOS Groups

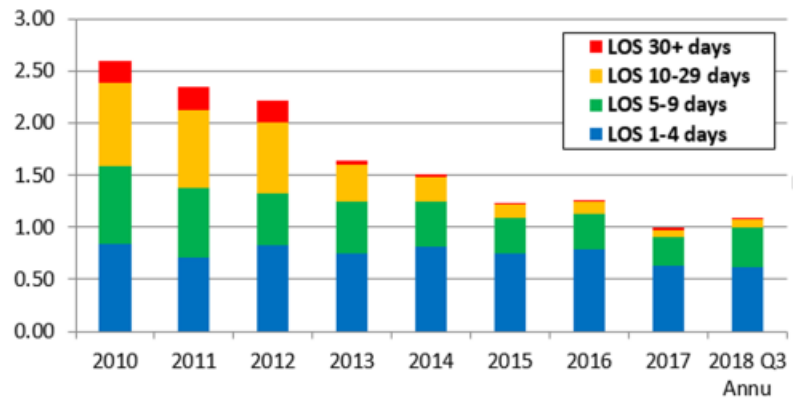

*Adjusted for Unique Patients*

Supplement: S1 Fig — (PDF) [file pone.0236360.s003.pdf]
