# Supplementary material for: Utilization, financial outcomes and stakeholder perspectives of a re-organized adult sickle cell program
Source: PLoS One. 2020 Jul 24;15(7):e0236360. doi: 10.1371/journal.pone.0236360 (PMC7380627; doi:10.1371/journal.pone.0236360)

S2 Table

YNHH Utilization by patients with only Medicaid insurance (Fig 4A).


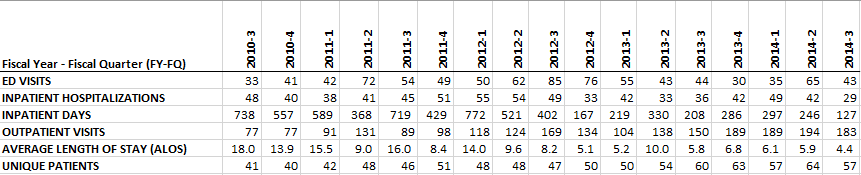


All other utilization by patients with only Medicaid insurance (Fig 4B).


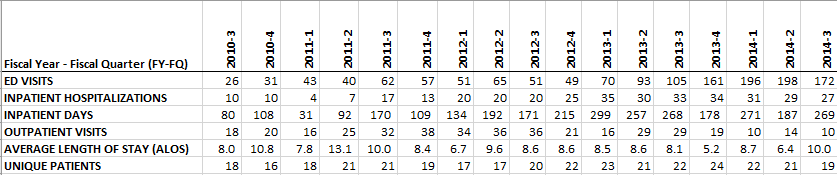

Supplement: S2 Table — (DOCX) [file pone.0236360.s002.docx]
