# Supplementary material for: Utilization, financial outcomes and stakeholder perspectives of a re-organized adult sickle cell program
Source: PLoS One. 2020 Jul 24;15(7):e0236360. doi: 10.1371/journal.pone.0236360 (PMC7380627; doi:10.1371/journal.pone.0236360)

S1 Document

Comparison of utilization at YNHH and a comparator institution.

The table shows total emergency department visits, inpatient discharges, outpatient visits, unique medical record numbers, total inpatient days, and average length of stay per quarter from 2010-1 through 2014-3 at YNHH (1) and a comparator institution (2).

Table.


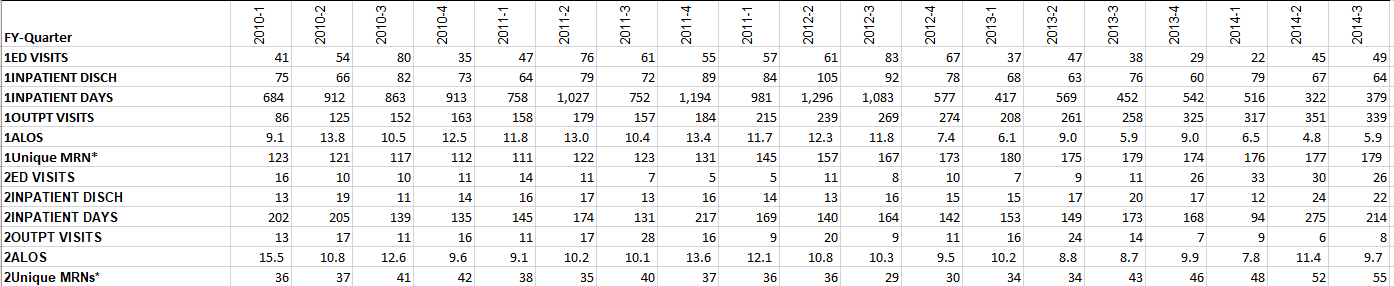


Inpatient days per quarter before and after the arrival of the medical director at YNHH and a comparator institution were compared using difference in differences and interrupted time series analyses in order to assess whether changes at YNHH represented a regional phenomenon potentially unrelated to the YNHH program re-organization. Analyses were done using Stata statistical software package V15.1.

YNHH had an average of 930.6 inpatient days (95% CI 860.9-1000.3) per quarter before and 471.8 inpatient days (95% CI 386.4-557.1) after. The comparator institution had an average of 162.0 inpatient days (95% CI 92.3-231.7) before and 171.0 (95% CI 85.6-256.4) after.  Difference in differences analysis showed that YNHH had change of 467.8 (95% CI 632.7 -312.0) fewer admissions compared to the comparator institution (p<0.001).

Interrupted time series analysis showed that following the arrival of the new medical director, YNHH had 65.2 fewer inpatient days per quarter than the comparator institution (95% CI 116.4 – 13.9, p=0.01).


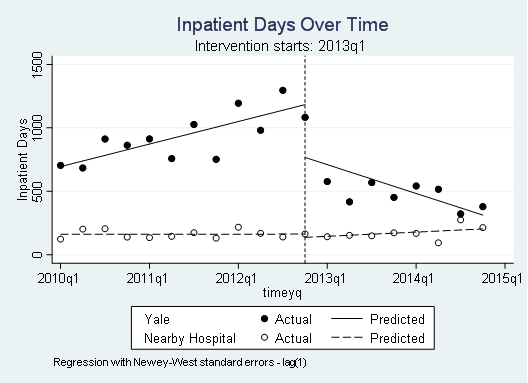

Supplement: S1 Document — (DOCX) [file pone.0236360.s004.docx]
