# Supplementary material for: Utilization, financial outcomes and stakeholder perspectives of a re-organized adult sickle cell program
Source: PLoS One. 2020 Jul 24;15(7):e0236360. doi: 10.1371/journal.pone.0236360 (PMC7380627; doi:10.1371/journal.pone.0236360)
Supplement: S2 Document — (DOCX) [file pone.0236360.s005.docx]

**S2 Document**

Adult Sickle Cell Unit Nurses Survey

**Q1. PCA improved pain management.**

1 2 3 4 5

Strongly Disagree Disagree Uncertain Agree Strongly Agree

**Q2. Tiered oral dosing improved pain management**

1 2 3 4 5

Strongly Disagree Disagree Uncertain Agree Strongly Agree

**Q3. Patient satisfied with PCA**

1 2 3 4 5

Strongly Disagree Disagree Uncertain Agree Strongly Agree

**Q4. Patient satisfied with tiered oral dosing**

1 2 3 4 5

Strongly Disagree Disagree Uncertain Agree Strongly Agree

**Q5. PCA pumps reduce nurses' time spent in medication administration activities**

1 2 3 4 5

Strongly Disagree Disagree Uncertain Agree Strongly Agree

**Q6. Noncompliant behavior by patients has been significantly reduced with the**

**implementation of integrated care plans.**

1 2 3 4 5

Strongly Disagree Disagree Uncertain Agree Strongly Agree

**Q7. Nurses are better equipped to deal with escalating patients after having the education**

**session on de-escalation.**

1 2 3 4 5

Strongly Disagree Disagree Uncertain Agree Strongly Agree

**Q8. Interdisciplinary rounds have improved nurses' knowledge about each patient's goals**

**of care.**

1 2 3 4 5

Strongly Disagree Disagree Uncertain Agree Strongly Agree

**Q9. Interdisciplinary rounds have improved nurses' involvement in goals of care decisions.**

1 2 3 4 5

Strongly Disagree Disagree Uncertain Agree Strongly Agree

**Q10. Interdisciplinary rounds have improved patients' involvement in goals of care**

**decisions.**

1 2 3 4 5

Strongly Disagree Disagree Uncertain Agree Strongly Agree

**Q11. Interdisciplinary rounds have improved patients' abilities to verbalize their goals of**

**care.**

1 2 3 4 5

Strongly Disagree Disagree Uncertain Agree Strongly Agree

**Q12. The SCD program/ unit including specialized staff education and training provide**

**nurses the ability to successfully care for patients with SCD.**

1 2 3 4 5

Strongly Disagree Disagree Uncertain Agree Strongly Agree

**Q13. Inpatient-Outpatient cross coverage by APRNs improves patients continuity of care.**

1 2 3 4 5

Strongly Disagree Disagree Uncertain Agree Strongly Agree
