# Supplementary material for: Utilization, financial outcomes and stakeholder perspectives of a re-organized adult sickle cell program
Source: PLoS One. 2020 Jul 24;15(7):e0236360. doi: 10.1371/journal.pone.0236360 (PMC7380627; doi:10.1371/journal.pone.0236360)
Supplement: S3 Document — (DOCX) [file pone.0236360.s006.docx]

S3 Document

ED Physician Assessment Survey Questions

Q1: Since program reorganization, when caring for sickle cell disease patients, my ability to do my job is ... *

Q2: Since program reorganization, when caring for sickle cell disease patients, my job related stress is... *

Q3: Since program reorganization, when caring for sickle cell patients, my job related satisfaction is... *

Q4: Do you think the number of ED visits by adults with sickle cell disease has changed since the re-organization? #

Q5: Do you think the number of ED visits by adults with sickle cell disease has changed since the re-organization? #

Q6: Do you think the ED length of stay for sickle cell patients has changed since re-organization? &

Q7: Do you think patients with sickle cell disease get better or worse care since reorganization? *

Q8: Overall, do you think reorganization of program has been fair to sickle cell patients? @

Q9: Do you rely on the use of Sickle Cells Plans in the FYI section of Epic? (Yes/No)

Q10: Sometimes the recommended opioid dose in the sickle cell plans is very high.  Are you comfortable with these plans with very high doses? !

Q11: Do Adults with sickle cell disease receive more aggressive pain treatment then adults with other painful conditions? %

Q12: Would You recommend Yale New Haven Hospital and its clinics to acquaintances with sickle cell disease? +

Likert scales

* Much Worse, Worse, No Change, Better, Much Better

# Many Fewer, Fewer, No Change, More, Many More

& Much Longer, Longer, No Change, Shorter, Much Shorter

@ Very Unfair, Unfair, Neither Fair nor Unfair, Fair, Very Fair

! Very Uncomfortable, Uncomfortable, Comfortable, Very Uncomfortable

% Much Less Aggressive, Less aggressive, More Aggressive, Much More Aggressive

+ Definitely No, Probably No, Probably Yes, Definitely Yes
